# Outcome of Japanese Encephalitis Virus (JEV) Infection in Pediatric and Adult Patients at Mahosot Hospital, Vientiane, Lao PDR

**DOI:** 10.4269/ajtmh.20-0581

**Published:** 2020-12-21

**Authors:** Mayfong Mayxay, Phouvieng Douangdala, Chanthala Vilayhong, Koukeo Phommasone, Vilada Chansamouth, Manivanh Vongsouvath, Sayaphet Rattanavong, Ko Chang, Onanong Sengvilaipaseuth, Anisone Chanthongthip, Soulignasack Thongpaseuth, Paul N. Newton, Audrey Dubot-Pérès

**Affiliations:** 1 Lao-Oxford-Mahosot Hospital-Wellcome Trust Research Unit (LOMWRU), Microbiology Laboratory, Mahosot Hospital, Vientiane, Laos;; 2 Institute of Research and Education Development, University of Health Sciences, Ministry of Health, Vientiane, Laos;; 3 Nuffield Department of Medicine, Centre for Tropical Medicine and Global Health, University of Oxford, Oxford, United Kingdom;; 4 Luang Namtha Provincial Hospital, Luang Namtha, Laos;; 5 Unité des Virus Émergents (UVE: Aix-Marseille Univ-IRD 190-Inserm 1207-IHU Méditerranée Infection), Marseille, France

## Abstract

Although Japanese encephalitis virus (JEV) infection is an important cause of acute febrile illness in Lao PDR (Laos), patient outcome has not been evaluated. We prospectively followed up 123 JEV-infected patients (70 children < 15 years and 53 adults ≥ 15 years) admitted at Mahosot Hospital, Vientiane, from 2003 to 2013. Japanese encephalitis virus infection was diagnosed by the detection of anti-JEV IgM in cerebrospinal fluid and/or IgM seroconversion. Neurological sequelae were assessed using the Liverpool Outcome Score (LOS), total (maximum score = 75), and final (maximum score = 5). The median (interquartile range [IQR]) age of the patients was 12.0 (7.5–18.8) years, and 57% were male. The median (IQR) duration of patients’ follow-up was 4.5 (3.2–7.3) years. Of all patients, 10/123 (8.1%) died during hospitalization, and 13/123 (10.6%) died at home after discharge, giving a mortality of 18.7% (23/123) (33 [26.8%] patients were lost to follow-up). The frequency of neurological sequelae at the last follow-up was 61.2% (48.4% in adults and 69.4% in children, *P* = 0.135). The proportion of patients with severe and moderate functional impairment at the last follow-up was significantly higher in children (25%) than in adults (6.5%), *P* = 0.042. Half of the patients who were still alive at the last follow-up (67) and for whom LOS data were available (22) had improvements in their total and final LOS between discharge and the last follow-up. The total and final LOS at discharge were not significantly different between children and adults, but total LOS at the last follow-up was significantly higher in adults than in children (median [IQR]: 74.5 [73–75] versus 73.0 [73–75], *P* = 0.019).

## INTRODUCTION

Japanese encephalitis virus (JEV) infection is a mosquito-borne disease and the leading cause of viral encephalitis in Asia, particularly in rural and suburban areas where rice culture and pig farming coexist.^1,2^ Although symptomatic Japanese encephalitis (JE) is rare among those infected, the case fatality rate in Asia among those with encephalitis can be as high as 30%, with permanent neurologic or psychiatric sequelae in 30–50% of survivors.^3–6^

Although the Lao PDR (Laos) is situated in Southeast Asia where human JEV infection has been described as a public health problem, the extent of JEV infection in Laos was not known until recently. The first report of acute JEV infection in Laos was by Moore et al.,^7^ through detection of anti-JEV IgM in cerebrospinal fluid (CSF) among patients with suspected central nervous system (CNS) infections at Mahosot Hospital, Vientiane. Recent studies have confirmed JEV infection across Laos,^8–10^ more commonly in the north than in the south. Aubry et al.^11^ described JEV genotype 1 from the CSF of a patient from Vientiane Province. A prospective study to identify the etiology of CNS infections among patients admitted at Mahosot Hospital, Vientiane Capital, demonstrated JEV infection as one of main causes.^9^ In response to these data, the Lao government introduced JEV vaccination in 2013. Although the case fatality rate and neurological sequelae following JEV infection are high, no studies have been conducted to determine clinical outcomes among Lao patients and very few studies in Asia on the long-term consequences.^5,12–14^ Liverpool Outcome Score (LOS) is a simple and reliable tool for assessing disability resulting from JEV infection.^15^ Liverpool Outcome Score was used in previous studies to assess clinical improvement among JE patients during follow-ups.^5,14,16^

Patients with confirmed JEV infection were prospectively followed up in hospital and after discharge to identify and compare the mortality and neurological outcomes between pediatric and adult patients, to yield better understanding of the consequence of this infection and provide information for public and policy engagement for vaccination.

## MATERIALS AND METHODS

### Study design, duration, and site.

We conducted a prospective study to follow up the clinical outcome of the patients with JEV infections admitted to Mahosot Hospital, Vientiane, Laos, from January 2003 to December 2013. Mahosot is a 365-bed primary- to tertiary-care hospital that specializes in internal medicine and general pediatrics and has ∼1,200 admissions per month. This hospital, along with four other major hospitals (1,210 beds total) and local provincial and district hospitals, serves a population of ∼900,000 people, including the urban population of Vientiane city and surrounding farming communities of Vientiane Province, and less frequently, outlying provinces.

### Patient diagnosis and treatment.

Patients with clinical suspicion of CNS infection (altered consciousness or other neurologic findings) and without contraindications for lumbar puncture (LP) who gave written informed consent had LP performed. No formal definition for CNS infection was used; patient recruitment was at the discretion of the responsible physician, reflecting local clinical practice. Cerebrospinal fluid and/or sera were collected and submitted to a large panel of etiological tests, as previously described.^9^ Cerebrospinal fluid and serum analyses were performed at the Microbiology Laboratory of Mahosot Hospital. Cerebrospinal fluid and sera were tested using JE—Dengue IgM Combo ELISA kit (Panbio Inc., Brisbane, Australia, now Alere Inc., Waltham, MA, Cat. No. E-JED01C) for the detection of anti-JEV IgM following the manufacturer’s instructions. For CSF, a dilution 1:10 was used, as described.^7,9^ Confirmed JEV infection was defined as detection of anti-JEV IgM in CSF or IgM seroconversion between admission and follow-up serum samples and no other etiology detected. Patients with JEV infections were monitored and treated according to Mahosot Hospital treatment guidelines and by the attending ward physicians.

#### Inclusion criteria.

Patients who were admitted to Mahosot Hospital with neurological findings suggestive of CNS infection, who had a LP, who consent to participate, and for whom JEV infection was confirmed (see above) were included in the current study.

#### Clinical assessment on admission.

On admission, patients were examined by the attending ward doctors, and information was recorded in a standard case record form and then entered in an electronic database. Retrospective data analysis was performed to classify patients with clinical acute encephalitis syndrome (AES) following WHO criteria^17^: patients with acute fever and change in mental status (confusion, disorientation, coma, or inability to talk) and/or new onset of seizures (excluding simple febrile seizures).

### Patient follow-up and neurological assessment.

At 1 month following discharge, surviving consenting patients without full recovery were asked to come back for follow-up at Mahosot Hospital or were visited at home if the patients declined to come to the hospital. Those living in the remoter districts for whom hospital and home visits failed (patients missed appointment or were not at home for two consecutive visits) were contacted by telephone. One to three follow-ups (depending on patient neurological status, traceability, and accessibility) were undertaken for each patient. The number of follow-ups for each patient depended on the judgment of the study doctors who assessed the patients as to whether further follow-up was needed or when of the next follow-up was required based on the neurological status (if full recovery was found, then no further follow-up was arranged), traceability, and accessibility (if the patients were not traceable or accessible either by home visit and/or telephone following the two consecutive attempts, then further follow-ups were stopped). At the follow-up, consenting patients were interviewed with or without help and support from their family members as needed and neurologically examined by four study doctors (M. M., P. D., K. C., and K. P.) who were trained in neurological examination. Neurological sequelae were assessed using the LOS and final LOS at discharge and at each follow-up by these four study doctors.^15^ The LOS assesses basic motor and self-care skills, as well as simple cognitive and behavioral functions, through a series of 10 questions, which were posed to the parent or caregivers, and five observations of the child performing simple activities (see Table 1). For many questions, both child and adult interviewees were asked to compare their ability with his or her peers of similar age in the community. The patient received a score for each question based on the grading scale. The final outcome score for each subject was the lowest score received for any question. Scores range from five (full recovery) to two (severe sequelae likely to make children and adults dependent), with a score of one being death. The interpretation of each outcome score is given in Table 1.

**Table 1:** Liverpool Outcome Score used to assess neurological sequelae of the patients with Japanese encephalitis virus infection – Lewthwaite et al.^15^

Answer each question	Score
Ask the patient or caregiver the following questions	
Speech or communication	
Normal	**5**
Changed or reduced	**3**
Not speaking or communicating	**2**
Feeding	
Normal	**5**
Occasionally needs help	**3**
Not speaking or communicating	**2**
Leaving alone	
Can be left alone	**5**
Briefly in familiar environment	**3**
Cannot be left alone	**2**
Behavior	
Normal	**5**
Gets angry easily	**4**
Other behavioral problems	**4**
Severely abnormal	**2**
Recognition	
Normal	**5**
Some	**3**
None	**2**
School or working	
Back to normal at school or work	**5**
Not doing as well	**4**
Dropped a school grade or no longer attending school or work	**3**
Epilepsy/seizure	
No seizure and not on anti- epileptic drugs	**5**
No seizure and on antiepileptic drugs	**4**
Yes, has had seizure	**3**
Yes, seizure most days	**2**
Dressing	
Normal	**5**
Occasionally needs more help	**3**
Always needs more help than other children of the same age	**2**
Bladder and bowel control	
Normal	**5**
Occasionally needs more help or occasionally is incontinent	**4**
Need more help or is incontinent of bowel or bladder	**2**
Hearing	
Normal	**5**
Reduced in one or both ears	**4**
Cannot hear at all	**3**
Observation of the patient’s abilities	
Sitting	
Normal or independently	**5**
Needs help	**3**
Not at all	**2**
Standing up	
Normal or independently	**5**
Needs help	**3**
Not at all	**2**
Walking	
Normally	**5**
Abnormally but independently ± crutches/stick	**3**
Not able to walk	**2**
Hands on head	
Normal both hands	**5**
Abnormal one or both hands	**3**
Unable one or both hands	**2**
Picking up	
Normal pincer grasp both hands	**5**
Unable one hand or abnormal one hand or both	**3**
Unable both hands	**2**

LOS = Liverpool Outcome Score. Outcome score: lowest score for any single question (range, 2–5). Total LOS: all the individual scores added up (range, 33–75). Final LOS is the lowest number scored for any single question: 5: full recovery; 4: minor sequelae with no effect, or only minor effects on physical function, or personality change, or on medication; 3: moderate sequelae mildly affecting function, probably compatible with independent living; 2: severe sequelae, impairing function sufficient to make patient dependent; 1: death.

### Data analysis.

Data were entered into an Excel worksheet and transferred to STATA version 14.1 (StataCorp, College Station, TX) for analysis. Descriptive statistics (number, percentage, median, and interquartile range [IQR]) were used to describe the characteristics of the patients and other clinical features. Children and adults were compared. The Wilcoxon rank-sum test was used for continuous variables and the chi-square or Fisher exact test for categorical variables. A *P*-value < 0.05 was considered statistically significant.

### Ethical approval.

Ethical approval for this study was granted by the National Ethics Committee for Health Research (NECHR) of the Lao Ministry of Health and Oxford Tropical Research Ethics Committee (OxTREC).

## RESULTS

### Patient description.

During the study period, 123 patients were included. Of these patients, 119/123 (96.7%) were diagnosed based on detection of anti-JEV IgM in CSF samples, while another 4/123 (3.3%) patients were diagnosed by detection of anti-JEV IgM in serum samples only with IgM seroconversion between admission and follow-up serum samples; anti-JEV IgM ELISA tests in CSF were negative. Of all patients, 70/123 (56.9%) were male and the median (IQR) age was 12.0 (7.5–18.8) years; 70/123 (56.9%) patients were children (< 15 years) and 16/123 (13.0%) were < 5 years. Approximately two-thirds of the patients (87/123 [70.7%]) were of Lao Loum ethnicity, and the proportion of non–Lao Loum patients (Khmou and Hmong) was significantly higher in children (26/70 [37.1%]) than in adults (10/53 [18.9%], *P* = 0.027). Forty-six of 123 (37.4%) patients were from Vientiane Capital, and 77/123 (62.6%) were from other provinces—mainly from Vientiane Province (31/123 [25.2%]). Fifty-three/123 (43.4%) patients had pigs living near their housing; 26/53 (49.1%) adult patients were rice farmers/laborers. For all 123 included patients, the median (IQR) estimated distance between patients’ houses and rice fields was ∼0.25 (0.05–0.65) km.

### Clinical symptoms and signs on admission.

Among all 123 patients, the median (IQR) duration of fever on admission was 5 (4–7) days and no significant difference was observed between children (5 [4–6] days) and adults (5 [4–7] days), *P* = 0.526 (Table 2). No significant difference was observed in the frequency of the symptoms and signs on admission in children compared with adults, except for myalgia (21/63 [33.3%] versus 33/53 [62.3%], *P* = 0.002), diarrhea (6/70 [8.6%] versus 0/53 [0%], *P* = 0.029), and seizure or convulsion (50/70 [71.4%] versus 12/53 [22.6%], *P* < 0.001). Acute encephalitis syndrome presentation on admission was significantly more frequent in children (63/69 [91.3%]) than in adults (39/52 [75.0%], *P* = 0.015). In all 123 patients, the median (IQR) Glasgow Coma Scale score on admission was lower in children (11 [8–13.5]) than adults (13 [10–15], *P* = 0.087).

**Table 2:** Characteristics of all 123 included patients with confirmed Japanese encephalitis virus infection

Variable	Total (*n* = 123)	Children (*n* = 70)	Adult (*n* = 53)	*P*-value†
Demographic				
Age (year), median (IQR)	12.0 (7.5–18.8)	8.0 (5.0–10.2)	20.0 (17.1–32.1)	
Female	53 (43.1%)	30 (542.9%)	23 (43.4%)	
Symptoms				
Number of days of fever, median (IQR)	5 (4–7)	5 (4–6)	5 (4–7)	0.526
	(*n* = 123)	(*n* = 70)	(*n* = 53)	
Headache*	104 (88.9%)	59 (92.2%)	45 (84.9%)	0.212
	(*n* = 169)	(*n* = 64)	(*n* = 53)	
Vomiting	73 (59.4%)	40 (57.1%)	33 (62.3%)	0.567
	(*n* = 123)	(*n* = 70)	(*n* = 53)	
Myalgia*	54 (46.6%)	21 (33.3%)	33 (62.3%)	0.002
	(*n* = 116)	(*n* = 63)	(*n* = 53)	
Visual loss*	6 (4.9%)	3 (4.4%)	3 (5.7%)	0.528‡
	(*n* = 122)	(*n* = 69)	(*n* = 53)	
Diarrhea	6 (4.9%)	6 (8.6%)	0	0.029
	(*n* = 123)	(*n* = 70)		
Cough	18 (14.6%)	10 (14.3%)	8 (15.1%)	0.900
	(*n* = 123)	(*n* = 70)	(*n* = 53)	
Anorexia*	76 (61.8%)	43 (61.4%)	33 (62.3%)	0.925
	(*n* = 123)	(*n* = 70)	(*n* = 53)	
Signs				
Glasgow Coma Score, median (IQR)	12 (9–14)	11 (8–13.5)	13 (10–15)	0.087
	(*n* = 119)	(*n* = 68)	(*n* = 51)	
Irritability	74 (60.2%)	46 (65.7%)	28 (52.8%)	0.148
	(*n* = 123)	(*n* = 70)	(*n* = 53)	
Confusion	71 (57.7%)	38 (54.3%)	33 (62.3%)	0.375
	(*n* = 123)	(*n* = 70)	(*n* = 53)	
Drowsiness	102 (82.9%)	59 (84.3%)	43 (81.1%)	0.645
	(*n* = 123)	(*n* = 70)	(*n* = 53)	
Seizure or convulsion	62 (50.4%)	50 (71.4%)	12 (22.6%)	< 0.001
	(*n* = 123)	(*n* = 70)	(*n* = 53)	
Stiff neck	111 (90.2%)	63 (90.0%)	48 (90.6%)	0.917
	(*n* = 123)	(*n* = 70)	(*n* = 53)	
Limb weakness	36 (29.3%)	25 (35.7%)	11 (20.8%)	0.071
	(*n* = 123)	(*n* = 70)	(*n* = 53)	
Hepatomegaly	13 (10.6%)	7 (10.0%)	6 (11.3%)	0.813
	(*n* = 123)	(*n* = 70)	(*n* = 53)	
Splenomegaly	1 (0.8%)	0	1 (1.9%)	0.431‡
	(*n* = 123)	(*n* = 70)	(*n* = 53)	
Jaundice	4 (3.3%)	2 (2.9%)	3 (3.8%)	1.000‡
	(*n* = 123)	(*n* = 70)	(*n* = 53)	
Rash	4 (3.3%)	2 (2.9%)	2 (3.8%)	1.000‡
	(*n* = 123)	(*n* = 70)	(*n* = 53)	
Lymph node	2 (1.6%)	1 (1.4%)	1 (1.9%)	1.000‡
	(*n* = 123)	(*n* = 70)	(*n* = 53)	
Syndrome				
AES	102§ (84.3%)	63 (91.3%)	39 (75.0%)	0.015
	(*n* = 121)	(*n* = 69)	(*n* = 52)	

AES = acute encephalitis syndrome. Data shown as the number (%) unless indicated.

* Only ask for children ≥ 5 years. Children: < 15 years, adult: ≥ 15 years.

† Wilcoxon rank-sum test was used for continuous variables and the chi-square or Fisher exact test for categorical variables.

‡ Fisher’s exact test.

§ Nineteen patients were not classified as AES; 15 had meningitis: fever and neck stiffness; four did not meet the criteria for AES either meningitis: one had GCS < 15 but not fever, the three other had normal GCS, no neck stiffness or seizure, but they had other symptoms or signs such as headache, limb weakness, visual loss, vomiting, or myalgia. No significant difference in outcome was observed between the patients classified as non-AES and the patients classified as AES.

### Hematological and CSF parameters on admission.

Among all 123 patients, the median (IQR) total peripheral white cell count (per mm^3^) on admission was higher in children (13,000 [10,000–18,900]) than in adults (11,645 [7,820–15,900], *P* = 0.095), but the differential white cell counts (neutrophils and lymphocytes) were not significantly different between the two age-groups (Table 3). By contrast, the median (IQR) white cell count (per mm^3^) in CSF on admission was significantly higher in adults (150 [50–320]) than in children (50 [20–125], *P* < 0.001).

**Table 3:** Hematological and CSF parameters on admission in all 123 included patients with confirmed Japanese encephalitis virus infection

Variable	Total (*n* = 123)	Children (*n* = 70)	Adults (*n* = 53)	*P*-value*
Hematological parameters				
Hematocrit (%), median (IQR)	38.0 (35.0–42.0)	36.5 (35.0–40.0)	40.0 (35.5–45.0)	0.007
	(*n* = 105)	(*n* = 59)	(*n* = 46)	
White blood cell count (cells/mm^3^), median (IQR)	12,000 (9,300–17,500)	13,000† (10,000–18,900)	11,645 (7,820–15,900)	0.095
	(*n* = 109)	(*n* = 61)	(*n* = 48)	
Neutrophils (%), median (IQR)	81 (71.2–89.4)	81 (71.4–90.2)	82 (69.7–89.0)	0.789
	(*n* = 100)	(*n* = 57)	(*n* = 43)	
Lymphocytes (%), median (IQR)	16.2 (7.8–24.1)	17.0 (8.3–24.6)	14.5 (7.2–21.5)	0.757
	(*n* = 104)	(*n* = 60)	(*n* = 44)	
Glycemia (mmoL/L), median (IQR)	6.4 (5.5–8.2)	6.3 (5.5–8.7)	6.6 (5.5–7.9)	0.738
	(*n* = 122)	(*n* = 70)	(*n* = 52)	
CSF parameters				
Opening pressure (cmH_2_0), median (IQR)	20 (16–24.6)	18.0 (14–24)	22.0 (18.8–25)	0.013
	(*n* = 121)	(*n* = 69)	(*n* = 52)	
White cell count (cells/mm^3^), median (IQR)	75 (30–125)	50 (20–125)	150 (50–320)	< 0.001
	(*n* = 123)	(*n* = 70)	(*n* = 53)	
Neutrophils (%), median (IQR)	20 (6–52)	18 (9–52)	21 (5–50)	0.955
	(*n* = 123)	(*n* = 70)	(*n* = 53)	
Lymphocytes (%), median (IQR)	70 (28–88)	66 (28–87)	70 (28–90)	0.698
	(*n* = 123)	(*n* = 70)	(*n* = 53)	
Glucose (mmoL/L), median (IQR)	3.6 (2.5–4.5)	3.4 (2.4–4.5)	3.6 (2.6–4.5)	0.800
	(*n* = 106)	(*n* = 58)	(*n* = 48)	
Protein (g/L), median (IQR)	0.7 (0.3–1.1)	0.5 (0.3–1.0)	0.7 (0.5–1.2)	0.028
	(*n* = 106)	(*n* = 58)	(*n* = 48)	

CSF = cerebrospinal fluid. Data shown as the number (%) unless indicated. Children: < 15 years; adults: ≥ 15 years.

* Wilcoxon rank-sum test was used.

† NB: no children were aged < 2 years old.

### Patients’ follow-up and clinical outcomes.

Of all included patients, 10/123 (8.1%) died during hospitalization, and 13/123 (10.6%) died at home after discharge, giving a potential total mortality of at least 18.7% (23/123). Of those who died after discharge from hospital, for eight patients, the day of death was known with a median (range; IQR) interval after discharge of 17 (0–1,460; 0.5–913.5) days. All deaths were thought to be related to JEV infection, based on information given by patient relatives, except one patient who died in road traffic crash 3 years after discharge. The proportion of deaths was higher in children (30.8% [16/52]) than in adults (18.4% [7/38]), but without statistical significance *P* = 0.183 (Table 4). Excluding the “lost to follow-up” patients (33/123 [26.8%]), clinical outcome was available for 90/123 (73.2%) patients, 52 children and 38 adults (Table 4). Of those 90 patients, 23/90 (25.6%) died (in hospital or after discharge), 26/90 (28.9%) completely recovered, and 41/90 (45.6%) had some clinical sequelae. The proportion of patients with complete clinical recovery was higher in adults [5/38, [39.5%]), than in children, (11/52, [21.2%]), *P* = 0.059.

**Table 4:** Clinical outcomes of the 90 Japanese encephalitis virus–infected patients who were clinically assessed

Variable	Total (*N* = 90)	Children (*n* = 52)	Adults (*n* = 38)	*P*-value
Fatalities				
Died in hospital	10 (11.1%)	7 (13.5%)	3 (7.9%)	
Died at home after discharge	13 (14.4%)	9 (17.3%)	4 (10.5%)	
Total deaths	23 (25.6%)	16 (30.8%)	7 (18.4%)	0.183
Patients who were alive at the last follow-up				
Complete clinical recovery	26 (28.9%)	11 (21.2%)	15 (39.5%)	0.213
Minor sequelae (slight behavioral changes)	30 (33.3%)	16 (30.8%)	14 (36.8%)	
Moderate or with some sequelae	8 (8.9%)	7 (13.5%)	1 (2.6%)	
Severe sequelae	3 (3.3%)	2 (3.9%)	1 (2.6)	

Data shown as the number (%) unless indicated.

Children: < 15 years; adults: ≥ 15 years.

### Patients’ neurological outcomes and LOS.

**Figure 1.**
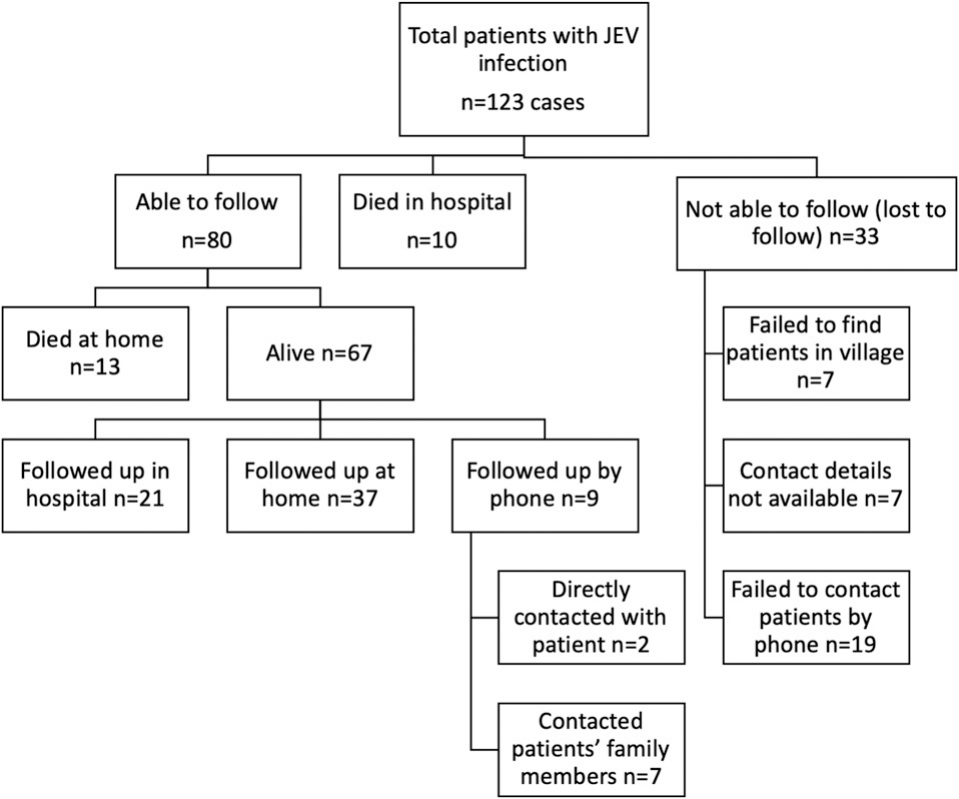
Flowchart of follow-up for the 123 Japanese encephalitis virus (JEV) patients included in the study.

Sixty-seven patients (36 children and 31 adults) were still alive at the last follow-up, 21/67 (31.3%) came back for the follow-up to hospital, 37/67 (55.2%) were followed up at home, and 9/67 (13.4%) were followed up by telephone calls (two directly with patients and seven with patients’ guardians or family members, see Figure 1. The median (IQR) duration of patients’ follow-up was 4.5 (3.2–7.3) years, with no significant difference between children and adults (*P* = 0.187). The follow-up and neurological outcomes based on LOS and final LOS of the 67 patients are shown in Table 5.

**Table 5:** Liverpool Outcomes Scores and duration of follow-up for 67 Japanese encephalitis virus–infected patients who were still alive at the last follow-up

Variable	Total (*n* = 67)	Children (*n* = 36)	Adult (*n* = 31)	*P*-value
Duration of follow-up				
Duration of follow-up (months), median (IQR)	54.1 (38.8–88.6)	53.7 (33.6–89.1)	54.7 (40.4–86.6)	0.187
	(*n* = 66)	(*n* = 36)	(*n* = 30)	
< 1-year follow-up, *n* (%)	0	0	0	0.992
1- to < 2-year follow-up, *n* (%)	7 (6.1)	5 (7.5)	2 (4.2)	
2- to < 3-year follow-up, *n* (%)	21 (18.3)	13 (19.4)	8 (16.7)	
3- to < 4-year follow-up, *n* (%)	12 (10.4)	6 (9.0)	6 (12.5)	
4- to < 5-year follow-up, *n* (%)	16 (13.9)	8 (11.9)	8 (16.7)	
5- to < 6-year follow-up, *n* (%)	8 (7.0)	5 (7.5)	3 (6.3)	
6- to < 7-year follow-up, *n* (%)	13 (11.3)	8 (11.9)	5 (10.4)	
7- to < 8-year follow-up, *n* (%)	10 (8.7)	6 (9.0)	4 (8.3)	
8- to < 9-year follow-up, *n* (%)	6 (5.2)	4 (6.0)	2 (4.2)	
9- to < 10-year follow-up, *n* (%)	8 (7.0)	5 (7.5)	3 (6.3)	
≥ 10-year follow-up, *n* (%)	14 (12.2)	7 (10.5)	7 (14.6)	
Total and final LOS				
Total LOS at discharge, median (IQR)*	70.5 (62–75)	69.5 (62–75)	71.5 (57–75)	0.375
	(*n* = 22)	(*n* = 10)	(*n* = 12)	
Total LOS at the last follow-up, median (IQR)	74 (73–75)	73 (73–75)	74.5 (73–75)	0.019
	(*n* = 64)	(*n* = 34)	(*n* = 30)	
Final LOS at discharge, median (IQR)†	3 (2–5)	2.5 (2–5)	3.5 (2–5)	0.663
	(*n* = 22)	(*n* = 10)	(*n* = 12)	
Final LOS at the last follow-up, median (IQR)	4 (4–5)	4 (4–5)	4.5 (4–5)	0.052
	(*n* = 64)	(*n* = 34)	(*n* = 30)	
Details of LOS at the last follow-up (from interview)				
Speech or communication				0.561
Normal (5)	60 (93.8)	31 (91.2)	29 (96.7)	
Changed or reduced (3)	3 (4.7)	2 (5.9)	1 (3.3)	
Not speaking or communicating (2)	1 (1.6)	1 (2.9)	0	
Feeding				0.344
Normal (5)	63 (98.4)	33 (97.1)	30 (100)	
Occasionally needs help (3)	0	0	0	
Not speaking or communicating (2)	1 (1.6)	1 (2.9)	0	
Leaving alone				0.402
Can be left alone (5)	62 (96.9)	32 (94.1)	30 (100)	
Briefly in familiar environment (3)	1 (1.6)	1 (2.9)	0	
Cannot be left alone (2)	1 (1.6)	1 (2.9)	0	
Behavior				0.151
Normal (5)	26 (40.6)	11 (32.4)	15 (50.0)	
Gets angry easily (4)	38 (59.4)	23 (67.7)	15 (50.0)	
Other behavioral problems (4)	0	0	0	
Severely abnormal (2)	0	0	0	
Recognition				–
Normal (5)	64 (100)	34 (100)	30 (100)	
Some (3)	0	0	0	
None (2)	0	0	0	
School or working				0.004
Back to normal at school or work (5)	40 (62.5)	15 (44.1)	25 (83.3)	
Not doing as well (4)	20 (31.3)	15 (44.1)	5 (16.7)	
Dropped a school grade or no longer attending school or work (3)	4 (6.3)	4 (11.8)	0	
Epilepsy/seizure				0.630
No seizure and not on antiepileptic drugs (5)	61 (92.3)	32 (94.1)	29 (96.7)	
No seizure and on antiepileptic drugs (4)	3 (4.7)	2 (5.9)	1 (3.3)	
Yes, has had seizure (3)	0	0	0	
Yes, seizure most days (2)	0	0	0	
Dressing				0.402
Normal (5)	62 (96.9)	32 (94.1)	30 (100)	
Occasionally needs more help (3)	1 (1.6)	1 (2.9)	0	
Always needs more help than other children of the same age (2)	1 (1.6)	1 (2.9)	0	
Bladder and bowel control				0.402
Normal (5)	62 (96.9)	32 (94.1)	30 (100)	
Occasionally needs more help or occasionally is incontinent (4)	1 (1.6)	1 (2.9)	0	
Need more help or is incontinent of bowel or bladder (2)	1 (1.6)	1 (2.9)	0	
Hearing				0.482
Normal (5)	61 (95.3)	33 (97.1)	28 (93.3)	
Reduced in one or both ears (4)	3 (4.7)	1 (2.9)	2 (6.7)	
Cannot hear at all (3)	0	0	0	
Details of LOS (from observation)				
Sitting				0.344
Normal or independently (5)	63 (98.4)	33 (97.1)	30 (100)	
Needs help (3)	0	0	0	
Not at all (2)	1 (1.6)	1 (2.9)	0	
Standing up				0.344
Normal or independently (5)	63 (98.4)	33 (97.1)	30 (100)	
Needs help (3)	0	0	0	
Not at all (2)	1 (1.6)	1 (2.9)	0	
Walking				0.249
Normally (5)	61 (95.3)	31 (91.2)	30 (100)	
Abnormally but independently ± crutches/stick (3)	2 (3.1)	2 (5.9)	0	
Not able to walk (2)	1 (1.6)	1 (2.9)	0	
Hands on head				0.344
Normal both hands (5)	63 (98.4)	33 (97.1)	30 (100)	
Abnormal one or both hands (3)	1 (1.6)	1 (2.9)	0	
Unable one or both hands (2)	0	0	0	
Picking up				0.344
Normal pincer grasp both hands (5)	63 (98.4)	33 (97.1)	30 (100)	
Unable one hand or abnormal one hand or both (3)	1 (1.6)	1 (2.9)	0	
Unable both hands (2)	0	0	0	

LOS = Liverpool Outcome Score. Data shown as the number (%) unless indicated. Children: < 15 years; adult: ≥ 15 years.

* Significantly different with score at the last follow-up (*P* = 0.010).

† Significantly different with score at the last follow-up (*P* = 0.005).

The total LOS (maximum score = 75) and final LOS (maximum score = 5) at discharge from hospital were not significantly different between children and adults, but the total LOS score at the last follow-up was significantly higher in adults (median: 74.5 [IQR: 73–75]) than in children (73 [73–75]), *P* = 0.019. Liverpool Outcome Score improvement between discharge and the last follow-up was observed for half of the patients, total LOS improvement in 12/22 (54.5%) patients, and final LOS improvement in 11/22 (50.0%) patients. Three of the 22 (13.6%) had worse final LOS and 5/22 (22/7%) had worse total LOS at the last follow-up than at discharge. The mean total LOS and mean final LOS were significantly higher at the last follow-up than those at discharge from hospital (paired-*t* test *P* = 0.010 and *P* = 0.005).

Details of neurological assessment at the last follow-up, based on the interview component of the LOS assessment, demonstrated that the proportions of patients with normal speech or communication, feeding, ability of living alone, behavior, recognition, dressing, bladder and bowel control, and hearing were not significantly different between children and adults, except that the frequency of patients who were able to return to normal school or work was significantly lower in children (15/34 [44.1%]) than in adults (25/30 [83.3.7%]), *P* = 0.004. Neurological assessment at the last follow-up also found no significant differences in the percentage of patients with abnormal sitting, standing up, putting their hand on their head, walking, and picking up items between the age-groups.

## DISCUSSION

When excluding patients lost to follow-up, the mortality in this cohort of Lao patients with suspicion of CNS infection and serological evidence of JEV infection was high at ∼26% (23/90). Japanese encephalitis virus infection mortality is likely to depend on many factors, including disease severity at presentation, in-hospital care quality (facilities and clinicians’ skills), socioeconomic and demographic status of the patients, length of patients’ follow-up following discharge from hospital, and home care of the patients following discharge. Furthermore, difference in fatality rates could depend on whether deaths in-hospital or after discharge from hospital are reported.^4,18^ The in-hospital JEV infection mortality among children in Indonesia was 25%,^5^ 25% for both child and adult patients in Thailand,^3^ 15% for children in India,^19^ and it was as high as 43% for adult patients in India.^13^ By contrast, in China, mortality among children with JEV infection in hospital was only 9%, and in Sarawak, Malaysia, only 8% of children died during the acute phase of JEV infection.^16,20^ In our study, the in-hospital mortality was also low (∼8%) for both child and adult patients, but this could be because Lao families often take moribund patients home to die.

To understand the true mortality of the disease, it is vital to follow up the patients with JE after discharge from hospital. In this study, pediatric and adult mortality for patients with JE following hospital discharge was ∼16%, higher than that described in Nepal^14^ and China^12^ where the equivalent percentage was only 8% during 5- to 12-month and 6- to 27-year follow-up, respectively.

Approximately 30–50% of survivors from JE have significant neurological sequelae.^18^ The proportion of the patients with functional impairment or neurological sequelae after acute JEV infections depends on factors such as severity of the disease at presentation, patients’ demography and socioeconomic status, quality of post-discharge care, and duration of patients’ follow-up.^4,18^ In Laos, the median duration of the patients’ follow-up after discharge from hospital was 4.5 (range, 1.5–13) years, and we found that about two-thirds of the survivors had neurological sequelae at the last follow-up, and this was higher in children than in adults (*P* = 0.135). In Indonesia, 50% of children with JE had functional impairment 1 year after discharge from hospital,^5^ and among Nepalese children, 48% had sequelae after the 1 year follow-up.^14^ Neurological sequelae were found in 59% of Malaysian children 3–6 months after discharge from hospital.^20^ Neurological sequelae among pediatric and adult patients with JE in China one to 2 years after discharge from hospital were ∼22–31%,^12,21^ and ∼25% in Thailand after the 1-year follow-up.^3^ Although the percentage of neurological sequelae found in our study patients was higher than that in other countries, patients with severe sequelae were few (∼3%) as compared with 25% in Indonesian children,^5^ 23% in Vietnamese patients,^22^ and 31% in Malaysian children.^20^ The lower neurological sequelae grade of severity found in our study patients could be due to the longer duration of follow-up (median = 4.6 years) of the patients in our study than others or that the very severe cases had died in or shortly after discharge from hospital.

Previous studies have demonstrated that the LOS of patients may improve through time during follow-up.^5,14,16^ Rehabilitation or post-discharge care of the patients with sequelae at home is probably vital for the recovery of the patients. In our study, although we did not investigate post-discharge care of patients, the total LOS and the total final LOS were significantly improved at the last follow-up for half of patients compared with these measures at discharge, suggesting that rehabilitation of the patients at home could probably play an important role in improving patients’ sequelae. Trialing of rehabilitation interventions through family members in rural Laos could lead to improvements in outcome. Using LOS and final LOS in assessing neurological sequelae of the patients at the last follow-up, we found that many parameters that we assessed such as speech or communication, feeding, living alone, recognition, dressing, bladder or bowel control, hearing, sitting, standing up, putting their hands on their head, and picking up things were almost normal in both children and adults but that behavior, back to normal school or work, and walking remained problematic, particularly among children. For instance, the overall frequency of patients who had what was regarded as normal behavior was just ∼40% and less than two-thirds of all patients were back to school or work normally at the last follow-up. A high proportion of JE survivors with abnormal behavior and difficulties in returning to normal school or work have also been reported in many studies, such as those in Vietnam and Cambodia,^23^ China,^21^ India,^13^ and Nepal.^14^ This suggests that a longer follow-up of these patients is needed to assess recovery.

An important limitation of this study was that a significant number of patients (∼one fourth) were lost to follow-up, and a small proportion of the patients’ follow-up details was retrieved from telephone conversations with family members, risking less reliable information acquisition. Only a small proportion of the patients who survived had LOS and total LOS assessed in hospital just before discharge from hospital, making the comparison of these scores with those at the last follow-up less powerful because of small paired sample sizes. We also cannot be sure that the deaths of patients during follow-up were direct consequences of JEV infection. Finally, the patients included in the study were admitted to hospital over a 10-year period, leading to variable lengths of follow-up and making impossible patient examination by a single doctor.

In conclusion, at least 1/5 of Lao inpatients with JEV infection died and nearly 2/3 of the survivors had neurological sequelae (half of them with minor sequelae) after a median duration of follow-up of 4.6 years. Sequelae were more common in children than in adults, but with approximately half of patients improving during follow-up. A longer follow-up of the survivors with sequelae is needed, and the economic impact should be assessed and rehabilitation interventions trialed.
